# Vascularized medial femoral condyle graft for nonunion after failed radiolunate arthrodesis

**DOI:** 10.1080/23320885.2018.1544848

**Published:** 2019-01-22

**Authors:** Akito Nakanishi, Shohei Omokawa, Kenji Kawamura, Takamasa Shimizu, Yasuhito Tanaka

**Affiliations:** a Department of Orthopedic Surgery, Nara Medical University, Kashihara city, Japan;; b Department of Hand Surgery, Nara Medical University, Kashihara city, Japan

**Keywords:** Vascularized bone graft, medial femoral condyle flap, radiolunate arthrodesis, nonunion, osteoarthritis

## Abstract

Since the medial femoral condyle flap was originally described in 1989, the indications for use of this versatile flap as a graft have broadened. We used this procedure in a patient with nonunion after failed arthrodesis of the radiolunate joint. Early bone union was achieved, with marked postoperative improvement in VAS and DASH scores.

## Introduction

The medial femoral condyle flap was originally described as a corticoperiosteal flap for local use in 1989, and subsequently the indications for grafting using this flap have broadened [1–3]. However, to the best of our knowledge, there are no previous reports on application of this procedure for radiolunate arthrodesis. We used this approach in a patient with nonunion after failed arthrodesis of the radiolunate joint. The purpose of this article is to introduce the procedure of radiolunate arthrodesis using a vascularized medial femoral condyle flap and to assess the clinical and radiological outcomes in this case.

## Case report

**Figure 1.**
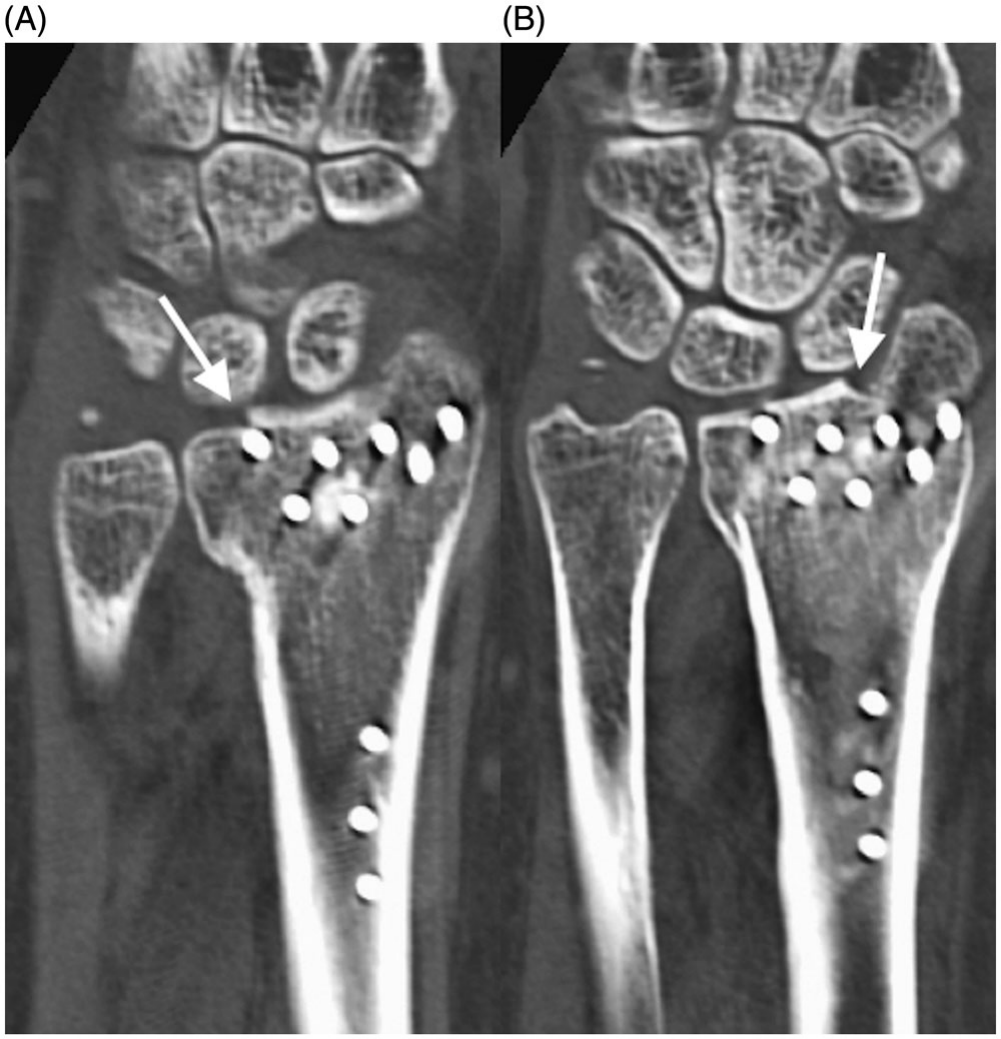
CT images showing an irregular articular surface due to poor reduction in the initial surgery.

A 32-year-old man fell from a high place and sustained a comminuted fracture in the right distal radius (AO classification type C1). He underwent open reduction and internal fixation using a volar plate at an emergency hospital. He visited the outpatient clinic of our department 24 months after the initial surgery because wrist pain had increased during his work. Radiographs showed an irregular articular surface of the distal radius due to poor reduction in the initial surgery (Figure 1). An examination revealed a restricted range of motion of the wrist joint (flexion/extension 40°/30°) and grip strength had decreased to 20% of the healthy side.

**Figure 2.**
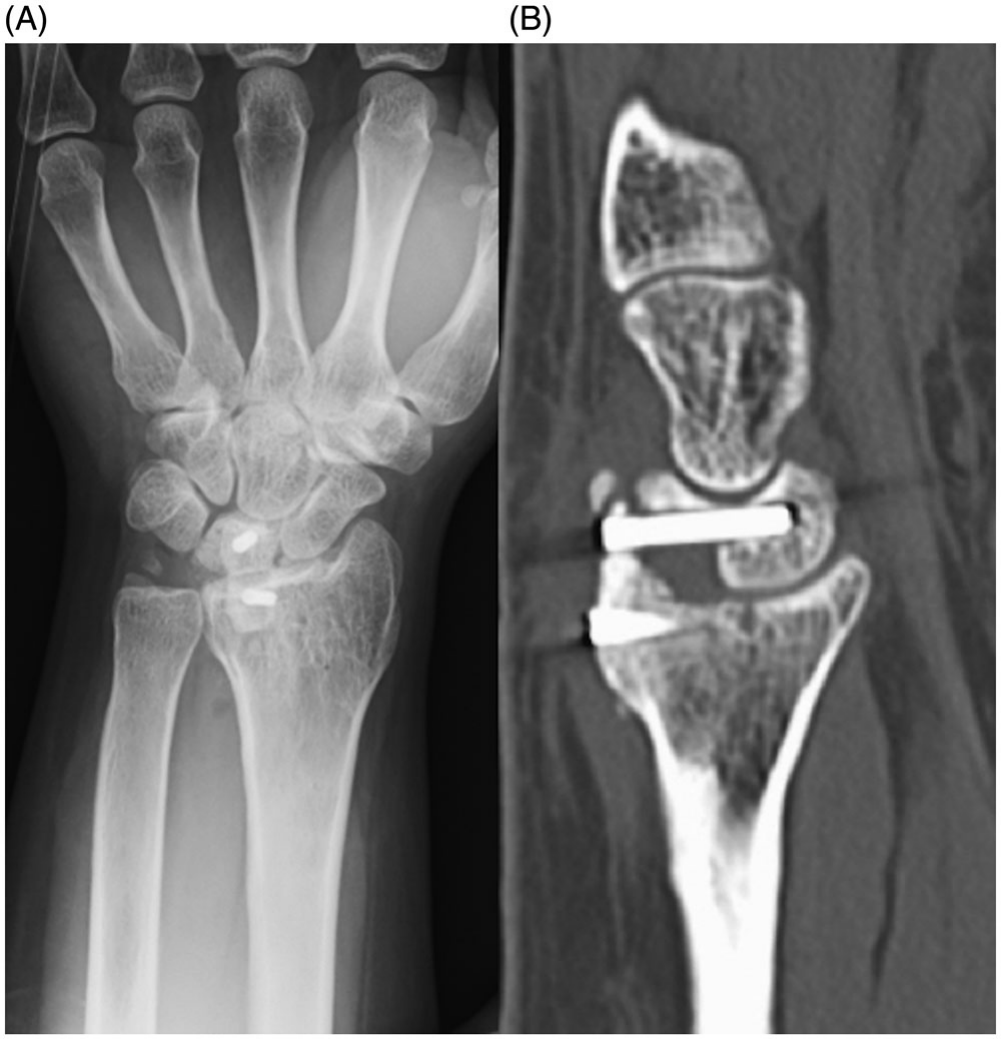
(A) Immediately postoperative radiograph of the radiolunate arthrodesis with the iliac crest. (B) Radiograph at 6 months after the first radiolunate arthrodesis showing nonunion.

**Figure 3.**
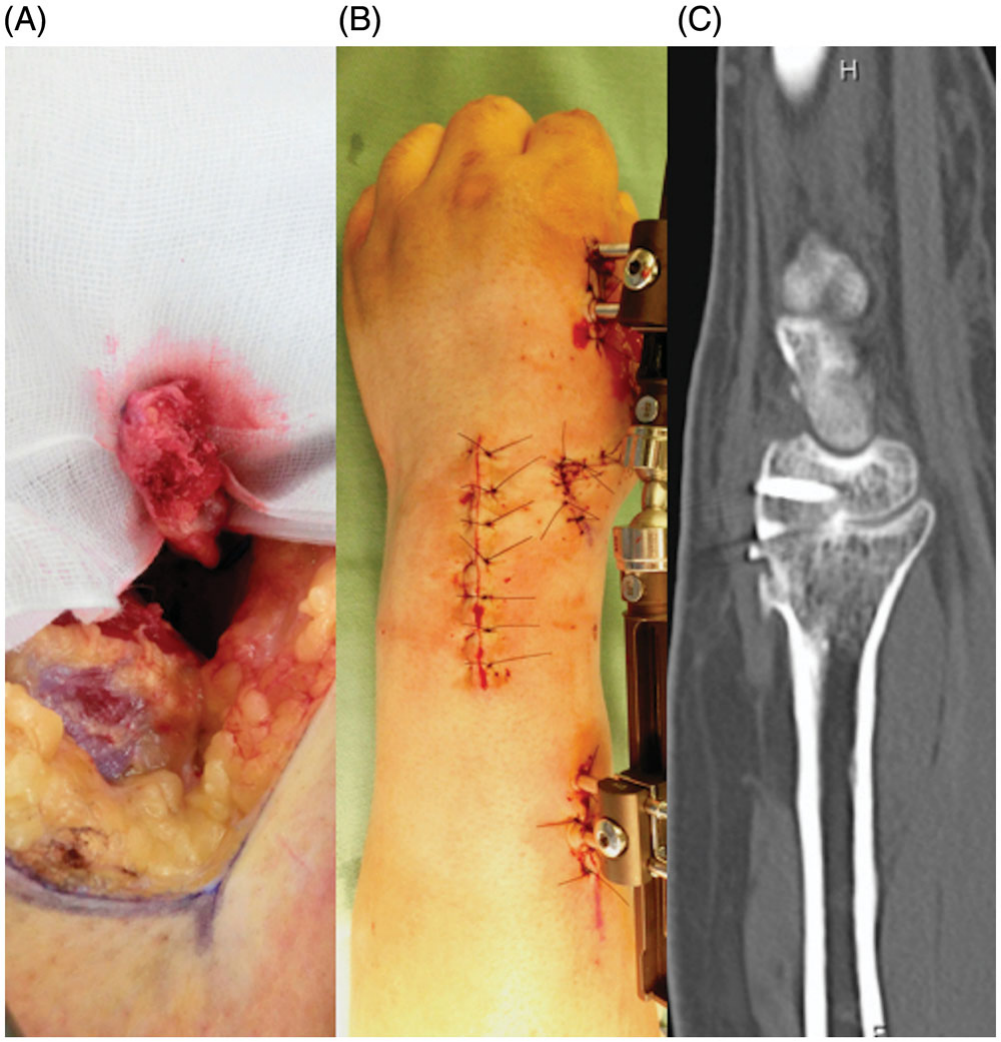
(A) Intraoperative photograph of the vascularized bone graft at the time of harvesting. (B) Immediately postoperative photograph of the wrist with the external fixation device. (C) At 3 months after the last surgery, a radiograph confirmed bony union.

**Figure 4.**
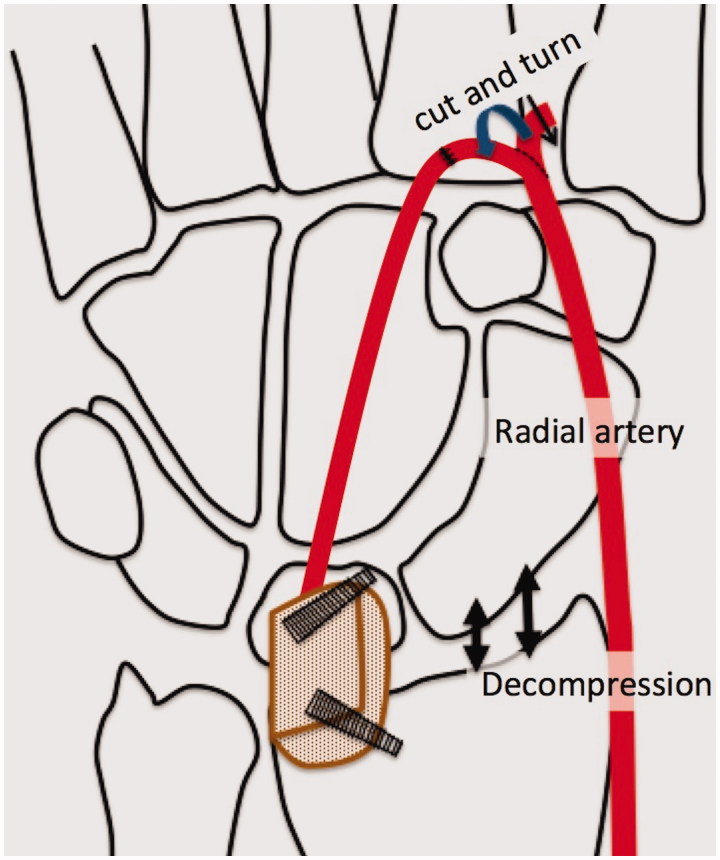
Diagrams of the surgical procedure. The bone graft was placed between the lunate and radius with a slight distraction of the lunate. End–to-end anastomosis was performed into the dorsal branch of the radial artery.

Removal of the plate and radiolunate arthrodesis with a free iliac bone graft was performed in our department (Figure 2(A)). The radiolunate fusion was done through a dorsal approach. However, the pain was still present at 6 months postoperatively, and radiography showed nonunion and loosening around the screws (Figure 2(B)). The patient was then treated with a vascularized bone graft from a medial femoral condyle bone flap (Figure 3). First, an external fixation device was used for the purpose of load reduction on the radiocarpal joint. Next, after exposing the radiocarpal joint, the fibrous damaged tissue including the iliac crest and screws at the nonunion site were removed. The damaged articular cartilage and subchondral bone were sufficiently excised to expose the underlying cancellous bone. The descending genicular vessels as the vascular pedicle were elevated with the periosteum and bone cortex and cancellous bone at the right medial femoral condyle bone (pedicle length 50 mm, vascularized graft segment 30 × 10 × 10 mm). The size of vascularized graft segment was decided considering intraoperative measurement of the bone defect. The bone graft was tailored to fit the radiocarpal joint and was placed between the lunate and distal radius with a slight distraction of the lunate, decompressing the scaphoid (Figure 4). Then, the bone graft was fixed with two headless compression screws at both the lunate and distal radius sites. End to end anastomosis into the dorsal branch of the radial artery and concomitant veins at the snuff box was performed.

An external fixation device was used for 4 weeks. This was due to a concern regarding the poor strength of femoral condyle bone with a thin cortex. The reason why we chose the external fixation instead of the dorsal spanning plate was to avoid worsening the blood circulation in the vascularized bone. There was no additional procedure performed at the donor site after harvesting the bone flap. A short arm cast was used for immobilization for 2 weeks after removal of the external fixation device. After cast removal, wrist and hand therapy was started, with the aim of regaining wrist and thumb mobilization. Ambulation was permitted starting on the first postoperative day. Complete bone union was achieved 8 weeks after the third surgery. At final follow up of 12 months postoperatively, the grip strength was restored to 92% of the healthy side, and the range of motion of the wrist and the VAS and DASH scores were improved (flexion/extension: 45°/50°, VAS: 85 to 5, DASH: 52 to 13). There was no morbidity such as pain at the donor site. The patient returned to unrestricted daily activity and his work 4 months after the third surgery.

## Discussion

Radiolunate arthrodesis, as reported by Chamay et al, is an effective procedure to relieve pain and retain some flexion/extension for treatment of degenerative radiolunate arthritis due to rheumatoid arthritis and distal radius fracture [4,5]. One disadvantage of radiolunate arthrodesis is non-union [6,7]. However, Ono reported good outcomes of radiolunate fusion using a vascularized radius graft for treatment of Bain’s grade 2A Kienböck disease with incongruity of the radiolunate joint [8].

In our case, it was difficult to achieve bone union in rearthrodesis surgery because the volume of lunate bone had decreased due to the first failed arthrodesis surgery. Thus, we chose a vascularized bone graft to obtain reliable bone union. We could not use a vascularized radius graft due to the initial complex distal radius fracture, and therefore, we had to use a free vascularized bone graft. Eventually, we chose a vascularized medial femoral condyle bone graft for the procedure. Our concern was insufficient strength of medial femoral condyle bone due to its flexibility, and for this reason we used an external fixation device for 4 weeks. Finally, the strength of the medial femoral condyle bone was sufficient and there were no problems at the arthrodesis site after bone union was obtained. In conclusion, this case shows that a vascularized medial femoral condyle bone graft is a good option in cases of nonunion after initial arthrodesis of the radiolunate joint.
